# Post COVID-19 mental health symptoms and quality of life among COVID-19 frontline clinicians: a comparative study using propensity score matching approach

**DOI:** 10.1038/s41398-022-02089-4

**Published:** 2022-09-09

**Authors:** Yan-Jie Zhao, Xiaomeng Xing, Tengfei Tian, Qian Wang, Sixiang Liang, Zhe Wang, Teris Cheung, Zhaohui Su, Yi-Lang Tang, Chee H. Ng, Sha Sha, Yu-Tao Xiang

**Affiliations:** 1 Unit of Psychiatry, Department of Public Health and Medicinal Administration, & Institute of Translational Medicine, Faculty of Health Sciences, University of Macau, Macao SAR, China; 2 Centre for Cognitive and Brain Sciences, University of Macau, Macao SAR, China; 3 Institute of Advanced Studies in Humanities and Social Sciences, University of Macau, Macao SAR, China; 4 The National Clinical Research Center for Mental Disorders & Beijing Key Laboratory of Mental Disorders, Beijing Anding Hospital & the Advanced Innovation Center for Human Brain Protection, Capital Medical University, Beijing, China; 5 School of Nursing, Hong Kong Polytechnic University, Hong Kong SAR, China; 6 Center on Smart and Connected Health Technologies, Mays Cancer Center, School of Nursing, UT Health San Antonio, San Antonio, TX USA; 7 Department of Psychiatry and Behavioral Sciences, Emory University, 12 Executive Park Drive NE, Suite 150, Atlanta, GA USA; 8 Atlanta VA Medical Center, 1670 Clairmont Road, Decatur, GA USA; 9 Department of Psychiatry, The Melbourne Clinic and St Vincent’s Hospital, University of Melbourne, Richmond, VIC Australia

**Keywords:** Scientific community, Human behaviour

## Abstract

**Background:**

The extent and severity of post-COVID-19 mental health symptoms among frontline clinicians are not clear. This study compared mental health symptoms (i.e., depression, anxiety, and insomnia symptoms) and global quality of life (QOL) after the first COVID-19 outbreak between the COVID-19 treating and non-COVID-19 treating frontline clinicians.

**Methods:**

This cross-sectional, comparative, convenient-sampling study was conducted between October 13 and 22, 2020, which was five months after the first COVID-19 outbreak in China was brought under control. The severity of depression, anxiety, insomnia symptoms, and global QOL of the clinicians were assessed using the Patient Health Questionnaire-9 items (PHQ-9), Generalized Anxiety Disorder Scale—7 items (GAD-7), Insomnia Severity Index (ISI), and the World Health Organization Quality of Life Questionnaire—brief version (WHOQOL-BREF), respectively. The propensity score matching (PSM) method was used to identify comparable COVID-19 treating and non-COVID-19 treating frontline clinicians. A generalized linear model (GLM) was used to assess the differences in PHQ-9, GAD-7, ISI, and QOL scores between the COVID-19 treating and non-COVID-19 treating frontline clinicians.

**Results:**

In total, 260 COVID-19 treating frontline clinicians and 260 matched non- COVID-19 treating frontline clinicians were included. Non-COVID-19 treating frontline clinicians experienced more frequent workplace violence (WPV) than the COVID-19 treating frontline clinicians (*χ*^*2*^ = 7.6, *p* = 0.006). COVID-19 treating frontline clinicians reported higher QOL compared to their non-COVID-19 treating frontline counterparts (b = 0.3, *p* = 0.042), after adjusting for WPV experience. COVID-19 treating and non- COVID-19 treating frontline clinicians reported similar PHQ-9, GAD-7, and ISI total scores (all *p* values > 0.05).

**Conclusion:**

This study did not reveal more severe post-COVID-19 mental health symptoms in COVID-19 treating frontline clinicians compared to non-COVID-19 treating frontline clinicians. It is possible that the implementation of timely and appropriate mental health, social and financial supports could have prevented the worsening of mental health symptoms among the COVID-19 treating frontline clinicians after the first COVID-19 outbreak in China.

## Introduction

The coronavirus disease 2019 (COVID-19) was first reported in the Hubei province of China at the end of 2019 [1] and was declared a pandemic by the World Health Organization (WHO) in March 2020 [2]. Since then, it has emerged in over 200 countries and territories [3]. Due to the relatively high death rate [4, 5], fast transmission, and lack of effective treatment of COVID-19, a vast number of clinicians volunteered to join the frontline efforts to combat COVID-19 in Hubei province in early 2020 [6–8].

During the first COVID-19 outbreak, due to a large number of infected patients and a shortage of personal protective gear, COVID-19 treating frontline healthcare staff experienced a very high heavy workload and risk of infection with the severe acute respiratory syndrome coronavirus 2 (SARS-CoV-2) [9], both of which could increase the risk of mental health problems. A previous study found that the prevalence of depression, anxiety, insomnia, and distress symptoms were 50, 45, 34, and 72%, respectively, among the COVID-19 treating frontline clinicians [10]. Several comparative studies also found that COVID-19 treating frontline healthcare workers were at higher risk of mental health consequences such as depression, anxiety, sleep problems, and trauma compared to non-COVID-19 treating frontline healthcare workers [10–14]. However, most studies were conducted at the early stage of the COVID-19 outbreak (before May 2020), and very few studies compared the post-COVID-19 mental health symptoms between COVID-19 treating and non-COVID-19 treating frontline clinicians.

Previous studies on the severe acute respiratory syndrome (SARS) outbreak found that COVID-19 treating frontline healthcare professionals experienced a higher risk of psychological problems such as distress, post-traumatic stress symptoms (PTSS), and burnout compared to their non-COVID-19 treating frontline counterparts even one year after the SARS outbreak [15]. The COVID-19 pandemic has persisted longer than expected and is likely to be endemic for some time [16, 17]; therefore, understanding the post-COVID-19 mental health symptoms among COVID-19 treating frontline clinicians is important [18] to reduce its long-term impact.

This study compared the post-COVID-19 mental health symptoms (i.e., depression, anxiety, and insomnia symptoms) and global QOL between the COVID-19 treating and non-COVID-19 treating frontline clinicians after the first COVID-19 outbreak in China.

## Methods

### Study setting and participants

This cross-sectional comparative study was conducted between October 13 and 22, 2020. This was considered a suitable period for investigating the post-COVID-19 mental health symptoms because the first outbreak was brought under control in China in May 2020 [19]. Following previous studies [20–22], to minimize the risk of COVID-19 transmission, participants were recruited and assessed using the online WeChat-based QuestionnaireStar program (Changsha Haoxing Information Technology Co., Ltd., Changsha, China) based on convenient sampling. A Quick Response (QR) code linked to the invitation and assessments was disseminated to all public hospitals in Beijing with the help of the Beijing Hospital Authority via WeChat, which is the most popular social network application in China, with around 1.2 billion monthly active users [23]. All clinicians who were working in public hospitals in Beijing needed to regularly report personal health status with WeChat during the pandemic; therefore, all were presumed to be WeChat users.

To be eligible, participants needed to meet the following criteria: (1) aged 18 years or older; (2) were clinicians working in public hospitals in Beijing during the COVID-19 pandemic; (3) provided online electronic informed consent. The study was conducted on a voluntary and confidential basis, and the study protocol was approved by the Ethics Committee of Beijing Anding Hospital.

### Data collection and assessment tools

A data collection form was used to collect demographic information, including age, gender, education level, occupation (e.g., doctors, nurses, medical technician, and others), personal annual income, and marital status. COVID-19 treating frontline clinicians were defined as those who volunteered to work in the medical support team in Hubei province in early 2020 (epicenter during the first COVID-19 outbreak in China) or directly cared for COVID-19 patients in local hospitals in Beijing since the COVID-19 outbreak. Clinicians who did not provide care for COVID-19 patients since during the pandemic were defined as the “non-COVID-19 treating frontline clinicians”.

The severity of depressive symptoms was assessed using the validated Chinese version of the Patient Health Questionnaire-9 items (PHQ-9), which consists of 9 items, and each scores from 0 (not at all) to 3 (almost every day) [24, 25]. A higher score represents more severe depression [26]. The psychometric properties of PHQ-9 Chinese version have been validated in Chinese populations [27, 28]. Participants were classified as “having depression” (depression hereafter) if their PHQ-9 total score was ≥5 [26].

The severity of anxiety symptoms was assessed using the validated Chinese version of the Generalized Anxiety Disorder Scale—7 items (GAD-7), which consists of 7 items, and each scores from 0 (not at all) to 3 (almost every day) [29]. The total score of GAD-7 ranges from 0 to 21, with a higher score indicating more severe anxiety [29]. The GAD-7 Chinese version has been validated in the Chinese population with good psychometric properties [30, 31]. Participants were classified as “having anxiety” (anxiety hereafter) if their GAD-7 total score was ≥5 [29].

The 7-item Insomnia Severity Index (ISI) was used to evaluate the severity of insomnia symptoms. Each item is scored from 0 (none/very satisfied) to 4 (very severe/very dissatisfied), with the total score from 0 to 28 [32]. The Chinese version of ISI showed satisfactory psychometric properties [33–35]. Participants were classified as “having insomnia” if their ISI total score was ≥8 [32].

Workplace violence (WPV) experienced by clinicians since the COVID-19 outbreak was evaluated with the 10-item Chinese version of the Workplace Violence Scale [36]. This scale covers various forms of violence, including four items on psychological violence (including but not limited to verbally abusing, disparaging, scolding, insulting, threats in person or by letter), and six items on physical violence (including physical attacks regardless of the consequence severity, as well as sexual violence) [36]. Each item is rated by a 4-point scale regarding the violence frequency ranging from 0 (never) to 3 (more than three times) [36]. Participants were considered as “having experienced WPV” if he or she reported any type of psychological or physical violence since the COVID-19 outbreak.

Global QOL was assessed with the first two items of the World Health Organization Quality of Life Questionnaire—brief version (WHOQOL-BREF), with a higher score representing higher QOL [37, 38]. The Chinese version of WHOQOL-BREF has been validated in the Chinese population with good psychometric properties [39, 40].

### Statistical analyses

#### Propensity score matching

Due to different demographic characteristics between the COVID-19 treating and non-COVID-19 treating frontline clinicians in this study, the optimal fixed ratio matching based on propensity scores was used to identify comparable COVID-19 treating and non-COVID-19 treating frontline clinicians with a matching ratio of 1:1.

The propensity score is the probability of a participant being assigned to a particular group (i.e., COVID-19 treating frontline clinicians in this study), calculated by a logistic regression model based on a given set of observed covariates (i.e., confounders) [41]. The propensity score matching procedure would match each participant in the COVID-19 treating frontline group with one non-COVID-19 treating frontline participant that has a similar value of the propensity score, thereby balancing the potential confounders between the two groups [41, 42]. The propensity score analysis could help reduce bias in research results by minimizing the confounding effects caused by unmatched demographic characteristics [42].

Confounders refer to variables that affect both the outcome variable and the grouping variable [43–45]; the potential confounders matched in the propensity score model are selected based on the variable-grouping relationships and the variable-outcome relationships [42, 44]. In this study, the variable-grouping relationships and the variable-outcome relationships were assessed using independent two-sample *t*-tests, Wilcoxon rank-sum tests, and chi-square tests as appropriate. Confounders were selected based on an expert consensus and the findings of previous studies in the propensity score model [42, 46, 47].

The balance of demographic characteristics after matching was assessed using standardized differences [42, 48–50]. To achieve a good matching balance, the absolute value of standardized difference was preferentially <0.1 [48, 51–53], with a minimum requirement of <0.25 [54, 55]. Mirror histograms were used to display the distributions of the propensity scores in the COVID-19 treating and the non-COVID-19 treating groups before and after matching.

#### Univariable analyses

In univariable analyses before and after matching, the demographic and clinical characteristics between the COVID-19 treating and non-COVID-19 treating clinicians were compared using independent two-sample *t-*tests, Wilcoxon rank-sum tests, and chi-square tests as appropriate. In the matched study sample, demographic characteristics that were significant in the univariable analyses were adjusted for in multivariable analysis models.

#### Multivariable analyses

In the matched study sample, the generalized linear model (GLM) was used to assess the differences in PHQ-9, GAD-7, ISI total scores, and QOL between the COVID-19 treating and the non-COVID-19 treating frontline clinicians while adjusting for the demographic characteristics that were still significant in the univariable analyses after matching.

All data analyses were conducted using Statistical Analysis System (SAS) OnDemand for Academics (SAS Institute Inc., Cary, NC, USA). The core SAS procedures implemented in this study were the *PSMATCH* procedure and the *GENMOD* procedure. Two-tailed *p* values less than 0.05 were considered statistically significant.

## Results

### Demographic and clinical characteristics of the whole sample

Altogether, 260 COVID-19 treating frontline clinicians and 1473 non-COVID-19 treating frontline clinicians participated in this survey and completed the assessment. COVID-19 treating and non-COVID-19 treating frontline clinicians were statistically different in age, sex, occupation composition, education level, and marital status (all *p* values < 0.05; Supplementary Table 1).

### Potential confounder selection for propensity score matching

According to the preliminary results of the variable-grouping and the variable-outcome relationships (Supplementary Tables 1, 2), occupation, education level, and marital status of the clinicians were selected as the potential confounders, all of which were matched in the propensity score model. Additionally, since age and sex were the most commonly used confounders in previous studies [44, 56–59] and considerably associated with mental health status and QOL [60, 61], age and sex were also selected for matching in the propensity score model.

### Propensity score matching

The propensity score matching procedure identified 260 comparable COVID-19 treating and non-COVID-19 treating frontline clinicians in each group, composing a matched study sample of 520 participants. The standardized difference in the propensity scores between the matched two groups was 0.03, indicating that the matching procedure achieved a good balance. The absolute standardized differences of age, sex, occupation, education level, and marital status were all <0.1 (Table 1), showing that a good matching balance was achieved in each potential confounder.

**Table 1:** Demographic and clinical characteristics of the matched sample (*N* = 520).

Variables	Total (*N* = 520)		Non-COVID-19 treating frontline clinicians (*N* = 260)		COVID-19 treating frontline clinicians (*N* = 260)		Univariable analyses			Standardized difference
	*n*	*%*	*n*	*%*	*n*	*%*	*χ*^*2*^	*df*	*p*	
Male gender	212	40.8	111	42.7	101	38.8	0.8	1	0.37	0.084
Occupation							1.9	3	0.59	0.016
Doctor	124	23.8	67	25.8	57	21.9				
Nurse	339	65.2	162	62.3	177	68.1				
Medical technician	48	9.2	26	10.0	22	8.5				
Others	9	1.7	5	1.9	4	1.5				
Education level							1.1	2	0.58	0.074
PhD	132	25.4	71	27.3	61	23.5				
Master	335	64.4	164	63.1	171	65.8				
Bachelor	53	10.2	25	9.6	28	10.8				
Personal annual income (CNY)							3.0	3	0.40	0.038
<200 thousand	336	64.6	169	65.0	167	64.2				
200–300 thousand	163	31.3	77	29.6	86	33.1				
300–500 thousand	16	3.1	11	4.2	5	1.9				
>500 thousand	5	1.0	3	1.2	2	0.8				
Marital status							0.6	2	0.73	0.063
Never married	174	33.5	85	32.7	89	34.2				
Married	329	63.3	165	63.5	164	63.1				
Divorced	17	3.3	10	3.8	7	2.7				
PHQ-9 total score of 5 and above	181	34.8	91	35.0	90	34.6	0.01	1	0.93	—
GAD-7 total score of 5 and above	155	29.8	75	28.8	80	30.8	0.2	1	0.63	—
ISI total score of 8 and above	91	17.5	48	18.5	43	16.5	0.3	1	0.56	—
Experienced WPV since COVID-19	53	10.2	36	13.8	17	6.5	7.6	1	**0.006**	—
	Mean	SD	Mean	SD	Mean	SD	*t/Z*	*df*	*p*	
Age	34.3	7.2	34.4	7.8	34.2	6.6	0.2	505.9^a^	0.84	−0.016
PHQ-9 total score	3.6	4.2	3.5	4.1	3.7	4.3	0.4	—^b^	0.69	—
GAD-7 total score	3.0	3.3	2.9	3.3	3.1	3.3	0.7	—^b^	0.50	—
ISI total score	4.0	5.1	3.9	5.3	4.1	5.0	0.5	—^b^	0.59	—
Global QOL	7.2	1.5	7.0	1.6	7.3	1.5	−2.5	518	**0.015**	—

Bold values indicates statistical significant *p* values (*p* < 0.05)*;* COVID-19 coronavirus disease 2019, *GAD-7* generalized anxiety disorder-7, *CNY* Chinese yuan, *df* degree of freedom, *PhD* degree of philosophy, *PHQ-9* patient health questionnaire-9, *QOL* quality of life, *ISI* insomnia severity index, *SD* standard deviation, *WPV* workplace violence.

^a^Satterthwaite corrected.

^b^Wilcoxon rank-sum test.

**Fig. 1:**
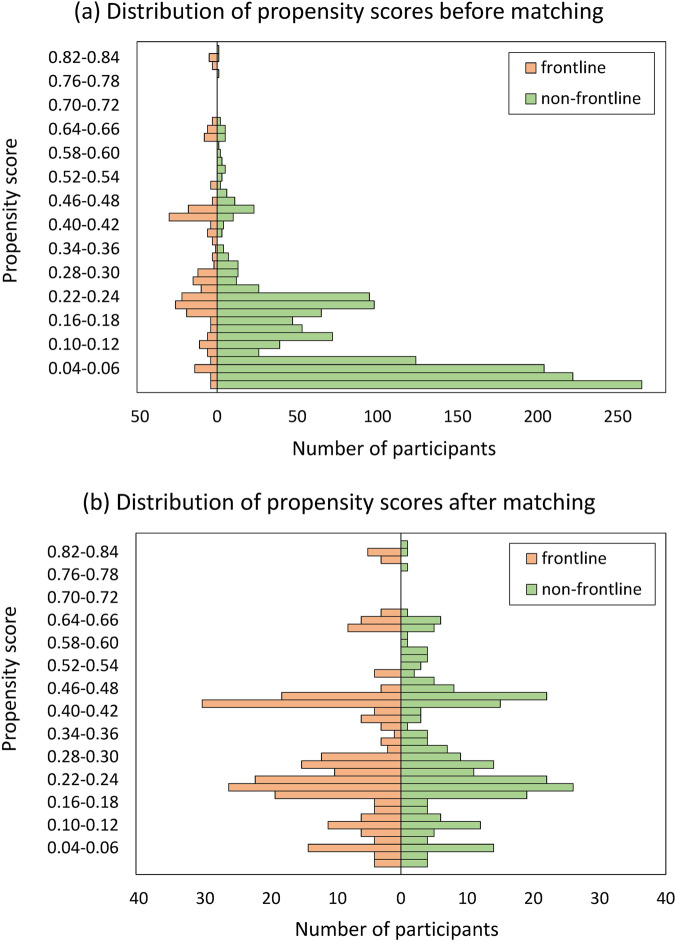
Distribution of propensity scores before and after matching.

The distributions of propensity scores before and after matching are shown in Fig. 1. Visual inspection of Fig. 1 found that the symmetry of propensity scores in the COVID-19 treating and non-COVID-19 treating frontline groups greatly improved after matching, further confirming the comparability between the two matched groups.

### Demographic and clinical characteristics of the matched sample

The demographic and clinical characteristics of the matched two samples are shown in Table 1. Univariable analyses revealed that the COVID-19 treating frontline clinicians and their matched non-COVID-19 treating frontline counterparts were comparable in age, sex, occupation, education level, personal annual income, and marital status (all *p* values > 0.05).

There were no significant differences between the COVID-19 treating and non-COVID-19 treating frontline clinicians in terms of the PHQ-9, GAD-7, and ISI total scores (all *p* values >0.05), while the COVID-19 treating frontline clinicians had higher QOL scores than the non-COVID-19 treating frontline group (*t* = −2.5, *p* = 0.015). Furthermore, the COVID-19 treating frontline clinicians experienced less frequent WPV than their non-COVID-19 treating frontline counterparts (13.8 vs. 6.5%; *χ*^*2*^ = 7.6, *p* = 0.006).

### Multivariable analyses

WPV during the COVID-19 outbreak was significantly associated with depression, anxiety, insomnia, and QOL scores (all *p* values < 0.05; Table 2). After adjusting for WPV, COVID-19 treating frontline clinicians still had higher QOL scores than their non-COVID-19 treating frontline counterparts (b = 0.3, 95% CI: 0.01–0.5, *p* = 0.042; Table 2). GLM analyses showed that work experience as COVID-19 treating frontline clinicians was not significantly associated with PHQ-9, GAD-7, and ISI total scores (all *p* values > 0.05; Table 2).

**Table 2:** Mental health symptoms and QOL and COVID-19 treating frontline work by GLM after controlling for the WPV (in the matched sample, *n* = 520).

Outcome variables	Corelated factors	*Wald χ*^*2*^	b	95% *CI*	*p*
PHQ-9 total score	COVID-19 treating frontline work	1.4	0.4	−0.3–1.2	0.23
	Experienced WPV	18.0	2.6	1.4–3.8	**<0.001**
GAD-7 total score	COVID-19 treating frontline work	1.0	0.3	−0.3–0.9	0.32
	Experienced WPV	10.9	1.6	0.6–2.5	**0.001**
ISI total score	COVID-19 treating frontline work	0.4	0.3	−0.6–1.2	0.55
	Experienced WPV	7.5	2.0	0.6–3.5	**0.006**
Global QOL	COVID-19 treating frontline work	4.1	0.3	0.01–0.5	**0.042**
	Experienced WPV	12.8	−0.8	−1.2– −0.3	**<0.001**

Bold values indicates statistical significant *p* values (*p* < 0.05)*; b* unstandardized regression coefficient, *CI* confidence interval, *COVID-19* coronavirus disease 2019, *GAD-7* generalized anxiety disorder-7, *GLM* generalized linear model, *PHQ-9* patient health questionnaire-9, *QOL* quality of life, *ISI* insomnia severity index, *WPV* workplace violence.

## Discussion

This comparative study found that the COVID-19 treating frontline clinicians had better QOL than their matched non-COVID-19 treating frontline counterparts after the first COVID-19 outbreak in China but did not find any significant group difference in terms of depression, anxiety, and insomnia symptoms.

To date, there have been no published studies that compared post-COVID-19 mental health symptoms and QOL between COVID-19 treating and non-COVID-19 treating frontline clinicians. Numerous studies on psychological responses to COVID-19, all of which were conducted before May 2020, found that COVID-19 treating frontline health workers were more likely to have lower QOL [62] and greater mental health problems, such as depression, anxiety, sleep problems, and PTSS, compared to their non-COVID-19 treating frontline counterparts [10–14, 63–66].

Following the first COVID-19 outbreak, COVID-19 treating frontline clinicians in China were provided with mental health, social and financial support. On February 22, 2020, the Central Leading Group for Responding to the COVID-19 Pandemic in China issued an announcement regarding support for COVID-19 treating frontline clinicians [67]. Measures to improve COVID-19 treating frontline clinicians’ welfare were proposed, including increased wages, opportunities for occupational promotion, improved work-related injury insurance, flexible rotating clinical work, additional support for families of COVID-19 treating frontline clinicians, and provision of psychological counseling services. As a result, these measures may have supported the COVID-19 treating frontline clinicians and offset the severity of post-COVID-19 mental health symptoms and improved their QOL. Additionally, some private-owned enterprises also provided financial supports for COVID-19 treating frontline clinicians [68, 69]. University students also volunteered to provide free tutorials for the children of COVID-19 treating frontline clinicians [70]. All the above-mentioned measures could have partly reduced the risk of post-COVID-19 mental health symptoms.

Several qualitative studies [71–74] found that COVID-19 treating frontline clinicians generally experienced three psychological phases during their work in the anti-pandemic frontline. The first phase is “duty and obligation that you cannot avoid”, in which clinicians volunteered to the frontline medical support team due to their occupational duties to take responsibility for patients’ health and well-being. The second phase is “physical and emotional exhaustion” due to heavy workload, unfamiliar working environment, wearing heavy personal protective equipment (PPE), loneliness, and fear of being infected. The third phase is “energy renewal, pride, and personal growth”, in which COVID-19 treating frontline clinicians gained psychological resilience, professional pride and recognition from colleagues, family members, and the public, gratitude from patients, and financial support from the government. Previous studies that reported worse mental health and decreased QOL among COVID-19 treating frontline clinicians compared to the non-COVID-19 treating frontline clinicians [10–14, 63–66] were mostly conducted during the initial COVID-19 outbreak (earlier than April 2020), which is aligned with the second phase of the psychological experience. In this study, the lack of severity in terms of post-COVID-19 mental health symptoms among the COVID-19 treating frontline clinicians could be partly because their experience was evolving into the third phase of the psychological experience.

Two possible factors could explain the lower QOL among non-COVID-19 treating frontline clinicians in this study. Non-COVID-19 treating frontline clinicians had less access to the medical information on the SARS-CoV-2 and less training on infection control measures compared to the COVID-19 treating frontline clinicians [75], which could reduce their confidence in combating the novel virus. Additionally, during the initial COVID-19 outbreak, the lack of PPE for clinicians not working in the anti-pandemic frontline may have led to an increased fear, negative psychological response, and decrease perceived QOL in non-COVID-19 treating frontline clinicians. One cross-sectional comparative study conducted in May 2020 in Malaysia found that non-COVID-19 treating frontline healthcare providers had experienced higher levels of depression, anxiety, and trauma compared to their COVID-19 treating frontline counterparts in response to the COVID-19 pandemic [76–78].

WPV was independently associated with more severe stress, anxiety, burnout [61, 79], and lowered global QOL [61]. The frequency of WPV experience in this study was considerably lower than that (1-year prevalence of WPV: 62.4%) reported in a meta-analysis conducted in China [79]. This may be attributed to several reasons. First, the timeframe of WPV experience in this study was “since the COVID-19 outbreak” (i.e., less than 1 year), while the meta-analysis used a 1-year timeframe [79]. Second, the meta-analysis was conducted in 2017. In recent years, the prevalence of WPV has gained considerable attention in China, and some effective measures have been adopted; for instance, mandatory security inspections before entering tertiary hospitals and lawsuits against perpetrators of WPV [80, 81]. Finally, in the guidelines for comprehensive measures to protect and care for COVID-19 treating frontline clinicians [67], creating a safer working environment for clinicians was listed as one of the priorities.

The strengths of this study included the matched demographic characteristics using the propensity score matching method. However, several limitations should be noted. First, due to the cross-sectional nature, this study could not make any inferences regarding the change in mental health symptoms over time in either COVID-19 treating or non-COVID-19 treating frontline clinicians. Second, the data were collected based on self-report; therefore, the possibility of recall bias could not be excluded. Third, for logical reasons, some factors associated with mental health symptoms, such as social support, were not measured. Fourth, this comparative study was based on convenient sampling, and the response rate could not be accurately estimated. Additionally, no data were collected on the general population, therefore, a direct comparison of mental health symptoms between the study sample and the general population during the same period could not be made.

In conclusion, this study did not find more severe post-COVID-19 mental health symptoms in COVID-19 treating frontline clinicians compared to non-COVID-19 treating frontline clinicians. It is possible that the implementation of timely and appropriate mental health, social and financial supports could have prevented the worsening of mental health symptoms among the COVID-19 treating frontline clinicians after the first COVID-19 outbreak in China.

## Supplementary information

supplementary tables
