# Supplementary material for: Post COVID-19 mental health symptoms and quality of life among COVID-19 frontline clinicians: a comparative study using propensity score matching approach
Source: Transl Psychiatry. 2022 Sep 9;12:376. doi: 10.1038/s41398-022-02089-4 (PMC9461449; doi:10.1038/s41398-022-02089-4)
Supplement: Supplementary file 1 — supplementary tables [file 41398_2022_2089_MOESM1_ESM.docx]

Supplementary Table 1. Demographic and clinical characteristics of the whole sample by COVID-19 treating frontline work (N=1,733)

| Variables | Total  (N=1,733) | | Non-COVID-19 treating frontline workers  (N=1,473) | | COVID-19 treating frontline workers  (N=260) | | Univariable analyses | | |
| --- | --- | --- | --- | --- | --- | --- | --- | --- | --- |
|  | *n* | *%* | *n* | *%* | *n* | *%* | *χ^2^* | *df* | *p* |
| Male gender | 441 | 25.4 | 340 | 23.1 | 101 | 38.8 | 28.9 | 1 | <0.001 |
| Occupation |  |  |  |  |  |  | 96.7 | 3 | <0.001 |
| Doctor | 482 | 27.8 | 425 | 28.9 | 57 | 21.9 |  |  |  |
| Nurse | 729 | 42.1 | 552 | 37.5 | 177 | 68.1 |  |  |  |
| Medical technician | 312 | 18.0 | 290 | 19.7 | 22 | 8.5 |  |  |  |
| Others | 210 | 12.1 | 206 | 14.0 | 4 | 1.5 |  |  |  |
| Education level |  |  |  |  |  |  | 20.4 | 2 | <0.001 |
| PhD | 472 | 27.2 | 411 | 27.9 | 61 | 23.5 |  |  |  |
| Master | 933 | 53.8 | 762 | 51.7 | 171 | 65.8 |  |  |  |
| Bachelor | 328 | 18.9 | 300 | 20.4 | 28 | 10.8 |  |  |  |
| Personal annual income (CNY) |  |  |  |  |  |  | 2.7 | 3 | 0.44 |
| <200 thousand | 1,099 | 63.4 | 932 | 63.3 | 167 | 64.2 |  |  |  |
| 200-300 thousand | 557 | 32.1 | 471 | 32.0 | 86 | 33.1 |  |  |  |
| 300-500 thousand | 64 | 3.7 | 59 | 4.0 | 5 | 1.9 |  |  |  |
| >500 thousand | 13 | 0.8 | 11 | 0.7 | 2 | 0.8 |  |  |  |
| Marital status |  |  |  |  |  |  | 55.3 | 2 | <0.001 |
| Never married | 311 | 17.9 | 222 | 15.1 | 89 | 34.2 |  |  |  |
| Married | 1,374 | 79.3 | 1210 | 82.1 | 164 | 63.1 |  |  |  |
| Divorced | 48 | 2.8 | 41 | 2.8 | 7 | 2.7 |  |  |  |
| PHQ-9 total score 5 and above | 577 | 33.3 | 487 | 33.1 | 90 | 34.6 | 0.2 | 1 | 0.62 |
| GAD-7 total score 5 and above | 542 | 31.3 | 462 | 31.4 | 80 | 30.8 | 0.04 | 1 | 0.85 |
| ISI total score 8 and above | 274 | 15.8 | 231 | 15.7 | 43 | 16.5 | 0.1 | 1 | 0.73 |
| Experienced WPV since COVID-19 | 127 | 7.3 | 110 | 7.5 | 17 | 6.5 | 0.3 | 1 | 0.60 |
|  |  |  |  |  |  |  |  |  |  |
|  | Mean | *SD* | Mean | *SD* | Mean | *SD* | *t/Z* | *df* | *p* |
| Age | 37.6 | 8.8 | 38.2 | 9.0 | 34.2 | 6.6 | 8.4 | 447.2^a^ | <0.001 |
| PHQ-9 total score | 3.6 | 4.1 | 3.6 | 4.1 | 3.7 | 4.3 | -0.1 | —^b^ | 0.89 |
| GAD-7 total score | 3.1 | 3.3 | 3.1 | 3.3 | 3.1 | 3.3 | -0.01 | —^b^ | 0.99 |
| ISI total score | 3.7 | 4.7 | 3.6 | 4.6 | 4.1 | 5.0 | 0.7 | —^b^ | 0.49 |
| Global QOL | 7.1 | 1.5 | 7.0 | 1.5 | 7.3 | 1.5 | -2.7 | 1731 | 0.006 |
| GAD-7: generalized anxiety disorder-7; CNY: Chinese yuan; df: degree of freedom; PhD: degree of philosophy; PHQ-9: patient health questionnaire-9; QOL: quality of life; ISI: insomnia severity index; SD: standard deviation; WPV: workplace violence.  a: Satterthwaite corrected; b: Wilcoxon rank sum test; | | | | | | | | | |

Supplementary Table 2. Demographic and clinical characteristics of the whole sample by depression (N=1,733)

| Variables | Total  (N=1,733) | | No depression  (N=1,156) | | Depression  (N=577) | | Univariable analyses | | |
| --- | --- | --- | --- | --- | --- | --- | --- | --- | --- |
|  | *n* | *%* | *n* | *%* | *n* | *%* | *χ^2^* | *df* | *p* |
| Male gender | 441 | 25.4 | 298 | 25.8 | 143 | 24.8 | 0.2 | 1 | 0.65 |
| Occupation |  |  |  |  |  |  | 9.6 | 3 | 0.022 |
| Doctor | 482 | 27.8 | 340 | 29.4 | 142 | 24.6 |  |  |  |
| Nurse | 729 | 42.1 | 460 | 39.8 | 269 | 46.6 |  |  |  |
| Medical technician | 312 | 18.0 | 206 | 17.8 | 106 | 18.4 |  |  |  |
| Others | 210 | 12.1 | 150 | 13.0 | 60 | 10.4 |  |  |  |
| Education level |  |  |  |  |  |  | 11.4 | 2 | 0.003 |
| PhD | 472 | 27.2 | 344 | 29.8 | 128 | 22.2 |  |  |  |
| Master | 933 | 53.8 | 597 | 51.6 | 336 | 58.2 |  |  |  |
| Bachelor | 328 | 18.9 | 215 | 18.6 | 113 | 19.6 |  |  |  |
| Personal annual income (CNY) |  |  |  |  |  |  | 3.9 | 3 | 0.28 |
| <200 thousand | 1,099 | 63.4 | 719 | 62.2 | 380 | 65.9 |  |  |  |
| 200-300 thousand | 557 | 32.1 | 379 | 32.8 | 178 | 30.8 |  |  |  |
| 300-500 thousand | 64 | 3.7 | 48 | 4.2 | 16 | 2.8 |  |  |  |
| >500 thousand | 13 | 0.8 | 10 | 0.9 | 3 | 0.5 |  |  |  |
| Marital status |  |  |  |  |  |  | 6.7 | 2 | 0.035 |
| Never married | 311 | 17.9 | 222 | 19.2 | 89 | 15.4 |  |  |  |
| Married | 1,374 | 79.3 | 908 | 78.5 | 466 | 80.8 |  |  |  |
| Divorced | 48 | 2.8 | 26 | 2.2 | 22 | 3.8 |  |  |  |
| GAD-7 total score 5 and above | 542 | 31.3 | 99 | 8.6 | 443 | 76.8 | 833.2 | 1 | <0.001 |
| ISI total score 8 and above | 274 | 15.8 | 41 | 3.5 | 233 | 40.4 | 392.3 | 1 | <0.001 |
| Experienced WPV since COVID-19 | 127 | 7.3 | 59 | 5.1 | 68 | 11.8 | 25.3 | 1 | <0.001 |
|  |  |  |  |  |  |  |  |  |  |
|  | Mean | *SD* | Mean | *SD* | Mean | *SD* | *t/Z* | *df* | *p* |
| Age | 37.6 | 8.8 | 37.5 | 9.1 | 37.8 | 8.2 | -0.7 | 1264.1^a^ | 0.49 |
| PHQ-9 total score | 3.6 | 4.1 | 1.2 | 1.4 | 8.4 | 3.5 | 34.7 | —^b^ | <0.001 |
| GAD-7 total score | 3.1 | 3.3 | 1.4 | 1.9 | 6.4 | 3.1 | 29.9 | —^b^ | <0.001 |
| ISI total score | 3.7 | 4.7 | 1.7 | 2.6 | 7.7 | 5.2 | 25.1 | —^b^ | <0.001 |
| Global QOL | 7.1 | 1.5 | 7.5 | 1.4 | 6.2 | 1.5 | 19.6 | 1731 | <0.001 |
| GAD-7: generalized anxiety disorder-7; CNY: Chinese yuan; df: degree of freedom; PhD: degree of philosophy; PHQ-9: patient health questionnaire-9; QOL: quality of life; ISI: insomnia severity index; SD: standard deviation; WPV: workplace violence.  a: Satterthwaite corrected; b: Wilcoxon rank sum test; | | | | | | | | | |
